# Electronic medical record-based deep data cleaning and phenotyping improve the diagnostic validity and mortality assessment of infective endocarditis: medical big data initiative of CMUH

**DOI:** 10.37796/2211-8039.1267

**Published:** 2021-09-01

**Authors:** Hsiu-Yin Chiang, Li-Ying Liang, Che-Chen Lin, Yi-Jin Chen, Min-Yen Wu, Sheng-Hsuan Chen, Pin-Hua Wu, Chin-Chi Kuo, Chih-Yu Chi

**Affiliations:** a Big Data Center, China Medical University Hospital, Taichung, Taiwan; b Division of Infectious Diseases, Department of Internal Medicine, China Medical University Hospital, Taichung, Taiwan; c Department of Medical Research, China Medical University Hospital, Taichung, Taiwan; d Department of Computer Science, National Tsing-Hua University, Hsinchu, Taiwan; e Kidney Institute and Division of Nephrology, Department of Internal Medicine, China Medical University Hospital, Taichung, Taiwan; f College of Medicine, China Medical University, Taichung, Taiwan

**Keywords:** Disease phenotyping, Electronic medical record, Infective endocarditis, International Classification of Diseases, Positive predictive value

## Abstract

**Background:**

International Classification of Diseases (ICD) code–based claims databases are often used to study infective endocarditis (IE). However, the quality of ICD coding can influence the reliability of IE research. The impact of complementing the ICD-only approach with data extracted from electronic medical records (EMRs) has yet to be explored.

**Methods:**

We selected the information of adult patients with discharge ICD codes for IE (ICD-9: 421, 112.81, 036.42, 098.84, 115.04, 115.14, 115.94, 424.9; ICD-10: I33, I38, I39) during 2005–2016 in China Medical University Hospital. Data extraction was conducted on the basis of the modified Duke criteria to establish a reference group comprising patients with definite or possible IE. Clinical characteristics and in-hospital mortality were compared between ICD-identified and Duke-confirmed cases. The positive predictive value (PPV) was used to quantify the IE identification performance of various phenotyping algorithms.

**Results:**

A total of 593 patients with discharge ICD codes for IE were identified, only 56.7% met the modified Duke criteria. The crude in-hospital mortality for Duke-confirmed and Duke-rejected IE were 24.4% and 8.2%, respectively. The adjusted in-hospital mortality for ICD-identified IE was lower than that for Duke-confirmed IE by a difference of 5.1%. The best PPV was achieved (0.90, 95% CI 0.86–0.93) when major components of the Duke criteria (positive blood culture and vegetation) were integrated with ICD codes.

**Conclusion:**

Integrating EMR data can considerably improve the accuracy of ICD-only approaches in phenotyping IE, which can improve the validity of EMR-based studies and their applications, including real-time surveillance and clinical decision support.

## 1. Introduction

The validity of electronic medical record (EMR)-based clinical research relies on accurate disease phenotyping. With advancements in computing technology and medical data extraction, methods for identifying multiple criteria–driven diagnoses of complex diseases should achieve higher accuracy than conventional International Classification of Diseases (ICD) code–based case identification schemes. Coding errors and inconsistencies in claims data have been reported in studies on infectious diseases, such as sepsis and health care–associated infections (HAIs) [1, 2]. Rhee et al. reported that the incidence of sepsis was overestimated when claims-based data were used (range, 8% to 12%) relative to estimates obtained using EMR-based clinical data (range, 5% to 6.5%) [1]. A systematic review suggested that ICD codes may be inaccurate for detecting HAIs other than *Clostridium difficile* or surgical site infections [2]. Moreover, the accuracy of ICD-based phenotyping is affected by variations in the policies and regulations of a health insurance system, the population covered by the healthcare system, and the coding behavior of clinicians, which consequently affect the interpretation and validity of clinical research findings [3–5]. However, few studies have investigated the impact of data curation on the identification of complex diseases requiring multiple clinical criteria. In this study, we used infective endocarditis (IE), a rare but lethal disease requiring multiple diagnostic criteria (i.e., the modified Duke criteria), to demonstrate how data extraction strategies improve the positive predictive value (PPV) of case identification beyond the ICD approach and how such strategies change mortality risk estimation.

## 2. Methods

### 2.1. Source population

The Big Data Center and the Office of Information Technology of China Medical University Hospital (CMUH) established the CMUH-Clinical Research Data Repository (CRDR) in 2017, which carefully verified and validated data from various clinical sources to unify trackable patient information generated during the healthcare process [6]. The CMUH–CRDR documented unified views of 2,660,472 patients who had sought care at the CMUH between January 1, 2003 and December 31, 2016. Patient information included data on administration and demography, diagnosis, medical and surgical procedures, prescriptions, laboratory measurements, physiological monitoring, hospitalization, and catastrophic illness status. The CMUH–CRDR has been linked to national population-based health-related databases, such as the National Death Registry, which are systematically maintained by the Health and Welfare Data Science Center of the Ministry of Health and Welfare. All patients enrolled in the CMUH–CRDR were followed up until December 31, 2016, or death, whichever occurred earlier.

### 2.2. Study population

This retrospective study included adult patients (≥18 years) with discharge ICD codes for IE (ICD-9: 421, 112.81, 036.42, 098.84, 115.04, 115.14, 115.94, 424.9; ICD-10: I33, I38, I39) [3, 4] who visited the CMUH between 2005 and 2016. The index date was the earliest date of IE diagnosis. Information on comorbidities (Supplemental Table 1), valvular replacement, microbiology reports, echocardiography reports, body temperature, and biochemical and urinalysis data was extracted from the CMUH–CRDR. We obtained mortality data by linking the CMUH-CRDR to Taiwan's National Death Registry.

### 2.3. Case validation

A research assistant (YJC) and an infectious disease specialist (LYL) systematically reviewed the medical charts and classified patients with IE diagnosis codes into definite, possible, or rejected groups according to the modified Duke criteria [7]. Using the Duke criteria as the reference standard, we evaluated the performance of ICD codes and their combinations with different EMR-derived clinical data in identifying patients with IE. We selected three clinical indicators, namely fever, positive blood culture, and cardiac vegetation confirmed through echocardiography reports, because they are objective and easily available, and because positive blood culture and vegetation evidence are the only two major components of Duke criteria, making them important indicators of IE. We used natural language processing (NLP) to extract keywords for the organism, Gram staining pattern, and antimicrobial susceptibility from microbiology reports. We used text mining to search for the keyword “vegetation” in echocardiography reports.

### 2.4. Statistical analysis

We analyzed the PPV for each case identification strategy. The study population was divided into true positive (Duke+ and case identification strategy+), true negative (Duke− and case identification strategy+), false positive (Duke− and case identification strategy+), and false negative (Duke+ and case identification strategy−). PPV was calculated by dividing the number of patients with IE confirmed using the Duke diagnostic criteria (definite or possible) by the total number of patients classified as IE based on different case identification strategies ( $\frac{TP}{TP+FP}$). The age-adjusted mortality was estimated using logistic methods [8]. Data were analyzed using SAS version 9.4 (SAS Institute Inc., Cary, NC). All analyses were two-sided, and the significance level was 0.05. The study was approved by the Big Data Center of CMUH and the Research Ethics Committee/ Institutional Review Board of CMUH (CMUH105-REC3-068).

## 3. Results

Of 593 adults with ICD codes for IE, only 336 (56.7%) met the modified Duke criteria (224 definite; 112 possible). Patients with Duke-confirmed IE were significantly younger and more likely to have hypertension, diabetes mellitus, chronic liver disease, and chronic kidney disease compared with those who did not meet the Duke criteria (Table 1). Among the patients with Duke-confirmed IE, 4.8% were diagnosed on the basis of minor criteria. Moreover, of the patients with Duke-confirmed IE, 70.8% had two positive blood cultures within 2 weeks of IE diagnosis and 58.3% yielded typical pathogens defined by the Duke criteria. Cardiac vegetation was detected in 88.4% of the patients with Duke-confirmed IE, but the detection rate dropped to 50.1% (297/593) in the entire population with ICD codes for IE (Table 1). Pyuria, hematuria, elevated erythrocyte sedimentation rate, or C-reactive protein was more frequently recorded among the patients with Duke-confirmed IE. The crude in-hospital mortality was threefold higher in the patients with Duke-confirmed IE (24.4%) than in Duke-rejected cases (8.2%; *P* < 0.0001). The mortality difference between the two groups persisted for at least 1 year after IE diagnosis.

We also evaluated the predictive performance for IE by combining three clinical criteria, namely fever, two positive blood cultures (PBCs), and echocardiographic evidence of vegetation, with ICD codes for IE. The age-adjusted in-hospital mortality for the study population (defined only by ICD) and reference standard (Duke-confirmed IE) were 15.9% and 21.0%, respectively (Table 2). When the Boolean operator “OR” was used to maximize the number of patients with IE identified using the case identification strategies, that is, the study population includes patients who had at least one of the three clinical criteria, the best PPV (0.90; 95% confidence interval [CI], 0.86–0.93) was achieved when PBC and vegetation were included. The corresponding age-adjusted in-hospital mortality was 21.8%, which approximated that of the reference group (Table 2). By contrast, when we applied the Boolean operator “AND” to maximize the specificity of the case identification strategies, that is, the study population includes patients who had two of the three or all three clinical criteria, the PPV was 1.00 whenever vegetation was included in the algorithm. The corresponding adjusted in-hospital mortality increased from 21.5% to 24.4%. When the case identification strategies defined only patients with concomitant fever, PBC, and vegetation as having IE, the adjusted in-hospital mortality was the highest at 24.4%.

Our original list of IE ICD codes included the ICD-9 code 424.9 (endocarditis valve unspecified) or ICD-10 code I38 (endocarditis, valve unspecified) that has not been used in some of the prior studies on IE [4, 5, 9, 10]. When we excluded patients with these two ICD codes, the PPV for the strategy applying only ICD codes (ICD-only strategy) increased to 0.83 (95% CI, 0.79–0.87), and the corresponding adjusted in-hospital mortality was lower by 1.2% relative to the reference strategy (Table 3). Introducing EMR-based phenotyping algorithms into the revised ICD-only approach did improve the PPV whenever PBC or vegetation was incorporated. However, 38 patients with Duke-confirmed IE were missed because they did not have the ICD code 424.9 or I38. These patients were more likely to be older and diagnosed on the basis of the Duke minor criteria compared with those having the ICD code 424.9 or I38 (Supplemental Table 2).

## 4. Discussion

This study revealed two notable findings. First, the cumulative incidence of IE was overestimated, but the mortality of IE was underestimated when only ICD codes were used as the estimation tool. Second, when EMR-based phenotyping was used, the accuracy of ICD-based phenotyping of IE could be improved. Despite its extensive implementation, the ICD-only approach should be reserved for claims databases.

For certain infectious diseases, such as sepsis and health care–associated infections, increasing bodies of evidence indicate that ICD codes may be inaccurate [1, 2]. In particular, the performance of an EMR-based phenotyping algorithm in retrospective databases is quantified by the PPV, although researchers must adjust for the negative predictive value or rare diseases with low prevalence and incidence, such as IE [11]. Our study identified only 56.7% of patients with discharge ICD codes, indicating that the diagnosis of IE met the Duke criteria (i.e., PPV, 0.57). Consistent with our findings, Fawcett et al. revealed that 44% and 56% of patients with IE ICD codes represented definite and possible IE, respectively, in two separate hospitals in the United Kingdom [10]. By contrast, a single-center study conducted in Canada demonstrated that the ICD-only approach could reach both high sensitivity and high specificity for definite or possible IE. However, the PPV based on ICD-10 was only 0.78 (95% CI 0.68–0.85), indicating that this approach cannot be generalized to other institutions [3]. In a study conducted in a US medical center, the PPV was 0.80 (95% CI 75.7–84.5) when an ICD extraction strategy similar to ours was used [4]. Although we could adjust the ICD search strategy (i.e., removing 424.9 or I38) to increase the PPV, a total of 38 patients with definite or possible IE were missed, leading to an underestimation of the disease burden and insufficient characterization of disease heterogeneity. Integrating EMR-based information can help avoid false-negative findings caused by the use of the highly sensitive ICD-only search strategy and can thus provide an accurate prevalence profile of IE.

Inaccurate coding may contribute to a moderate PPV and may be caused by clinicians' inexperience or attention to detail. For example, under Taiwan's National Health Insurance system, clinicians might upcode diagnoses to avoid refusal of reimbursement by health insurance agencies [12]. Moreover, patient factors constitute a major reason for upcoding. Aged individuals have a higher prevalence of valvular heart disease (VHD) and an increased risk of VHD-related IE compared with other individuals [13]. For example, the worsening of VHD-related murmurs might cause the misclassification of a minor criterion (VHD with regurgitation) into a major one (endocardial involvement), resulting in the over-estimation of IE cases [7]. The between-institution heterogeneity in the validity of ICD-based case identification approaches highlights the importance of in-house validation as a quality assessment strategy for clinical research conducted using EMRs.

Our study revealed that elderly patients with cardiovascular comorbidities tended to be assigned with IE-related ICD codes, indicating that misclassification bias can be differential with respect to mortality risk. This minimizes mortality risk underestimation in the ICD-only approach because studies that have used the ICD-only approach for IE identification have reported in-hospital mortality ranging from 14% to 20.4% [14–16]. By contrast, studies that have used the modified Duke criteria for final case identification have revealed slightly higher in-hospital mortality (ranging from 13% to 38.7%) [9, 16–24]. Although the discrepancy in mortality was not significant, it could affect the validity of the risk evaluation of potential factors, such as causative microorganisms and comorbidities. Researchers should appreciate the impact of case identification algorithms on variations in the risk of mortality due to IE in the literature. Comparison of mortality outcomes for IE that are not defined by the Duke criteria can be confounded by misclassification errors due to inadequate disease phenotyping. In our study, we observed that mortality associated with the three main clinical indicators of IE were different and that patients with PBC tended to have a higher probability of mortality than did those without PBC. Future research should evaluate whether variations in mortality arise from differences in the diagnostic components of the Duke criteria.

With the increased availability of EMR-based data, researchers can now maximize the potential of EMRs by using new computing technology, such as NLP, to improve the accuracy of case identification. Rhee et al. suggested that EMR-based clinical data provide more objective estimates in sepsis surveillance than do claims-based data [1]. Wei et al. also suggested that multiple EMR-based criteria afford higher identification performance than does a single criterion for a selected phenotype [25]. Our results demonstrate that the use of three EMR-based clinical criteria can considerably improve the PPV in identifying patients with definite or possible IE. Manually reviewing medical records to determine patients with IE on the basis of the modified Duke diagnostic criteria is a labor- and time-intensive process and requires trained personnel with clinical knowledge. By contrast, EMR-derived clinical criteria and ICD codes are mutually complementary and can be combined to automatically screen patients for IE in real time. In this study, the EMR-based algorithm identified cases that approximated the Duke-confirmed IE cases when we combined one of the two major components (i.e., PBC or vegetation) of the Duke criteria with ICD. Even when we incorporated a minor component of the Duke criteria, such as fever, with ICD, the identification performance was superior to that of the ICD-only approach. This combination approach can considerably reduce the burden of manual validation in conventional human-in-the-loop case identification processes.

This study has several limitations. First, the generalizability of our findings is limited due to the nature of a single-center setting. However, the differences in PPV and mortality arisen from data extraction strategies in EMR may be extrapolate to other databases and may highlight the importance of in-house data curation. Second, the misdiagnosis of IE was not explored. However, systematic screening of IE is not standard practice. In the future, the use of more advanced and updated NLP methodologies to systematically collect all components of the Duke criteria in EMRs will enable researchers to objectively compare the validity of ICD-only and EMR-driven phenotyping strategies.

## 5. Conclusion

In the era of EMR-driven phenotyping and knowledge discovery, integrating structured and unstructured data can considerably improve the accuracy of ICD-only approaches in phenotyping conditions such as IE, and therefore, improve the validity of EMR-based retrospective surveillance and cohort studies. In the future, automatically mapping multisource clinical data through EMRs to estimate patients' IE risk can facilitate efficient real-time case identification in clinical research and practice.

## Appendices

**Supplemental Table 1:** International Classification of Diseases, 9^th^ Revision, Clinical Modification (ICD-9-CM) diagnosis codes and ICD-10-CM diagnosis codes for defining comorbidities within 1 year of infective endocarditis diagnosis.

Comorbidities	ICD-9-CM	ICD-10-CM
Congestive heart failure	398.91, 402.01, 402.11, 402.91, 404.01, 404.03, 404.11, 404.13, 404.91, 404.93, 425.4–425.9, 428.x	I09.9, I11.0, I13.0, I13.2, I25.5, I42.0, I42.5-I42.9, I43.x, I50.x, P29.0
Diabetes mellitus	250.0–250.3, 250.8, 250.9, 250.4–250.7	E10.0, E10.l, E10.6, E10.8, E10.9, E11.0, E11.1, E11.6, E11.8, E11.9, E12.0, E12.1, E12.6, E12.8, E12.9, E13.0, E13.1, E13.6, E13.8, E13.9, E14.0, E14.1, E14.6, E14.8, E14.9, E10.2-E10.5, E10.7, E11.2-E11.5, E11.7, E12.2-E12.5, E12.7, E13.2-E13.5, E13.7, E14.2-E14.5, E14.7
Chronic liver disease	070.22, 070.23, 070.32, 070.33, 070.44, 070.54, 070.6, 070.9, 570.x, 571.x, 573.3, 573.4, 573.8, 573.9, V42.7, 456.0–456.2, 572.2–572.8	B18.x, K70.0-K70.3, K70.9, K71.3-K71.5, K71.7, K73.x, K74.x, K76.0, K76.2-K76.4, K76.8, K76.9, Z94.4, I85.0, I85.9, I86.4, I98.2, K70.4, K71.1, K72.1, K72.9, K76.5, K76.6, K76.7
Hypertension	401–405	I10–I15
Peripheral vascular disease	093.0, 437.3, 440.x, 441.x, 443.1–443.9, 447.1, 557.1, 557.9, V43.4	I70.x, I71.x, I73.1, I73.8, I73.9, I77.1, I79.0, I79.2, K55.1, K55.8, K55.9, Z95.8, Z95.9
Chronic kidney disease	582, 585, 586, 588, 583.0–583.7 ESRD 585 (Catastrophic illness)	ESRD: N18.5, N18.6, I12.0, I13.2, I13.11

**Supplementary Table 2:** Demographic and clinical characteristics of patients with infective endocarditis confirmed on the basis of Duke criteria (definite or possible).

Variables	Patients with Duke-confirmed IE (N = 336)		P value

	With ICD of 424.9 or I38 N = 298 (88.7%)	Without ICD of 424.9 or I38 N = 38 (11.3%)	
**Age** (year, median [Q1, Q3])	59.1 (46.22, 72.16)	71.79 (52.46, 79.13)	
18–64 years	186 (62.42)	14 (36.84)	0.003
≥65 years	112 (37.58)	24 (63.16)	
**Male**	183 (61.41)	20 (52.63)	0.30
**Comorbidities** a			
Congestive heart failure	79 (26.51)	15 (39.47)	0.09
Hypertension	100 (33.56)	14 (36.84)	0.69
Diabetes mellitus	102 (34.23)	8 (21.05)	0.10
Atrial fibrillation	54 (18.12)	6 (15.79)	0.72
Chronic liver disease	37 (12.42)	3 (7.89)	0.42
Chronic kidney disease	82 (27.52)	9 (23.68)	0.62
Peripheral vascular disease	10 (3.36)	2 (5.26)	0.55
**Duke criteria**			<0.0001
2 major	159 (53.36)	14 (36.84)	
1 major and 3–5 minor	46 (15.44)	5 (13.16)	
0 major and 5 minor	-	-	
1 major and 1–2 minor	85 (28.52)	11 (28.95)	
0 major and 3–4 minor	8 (2.68)	8 (21.05)	
0 major and 0–2 minor	-	-	
**Valve replacement surgery** b	53 (17.79)	4 (10.53)	0.26
**Days from admission to diagnosis**, median (Q1–Q3)	8 (1, 27)	9 (1, 23)	
**Blood culture**			
Two positive cultures within 14 days following IE diagnosis	216 (72.48)	22 (57.89)	0.06
Two positive cultures with typical pathogens ^c^	176 (59.06)	20 (52.63)	0.45
**Sonographic evidence of vegetation**	273 (91.61)	24 (63.16)	<0.0001
**Fever (≥ 38°C)**	151 (50.67)	26 (68.42)	0.04
**Urinalysis**, median (Q1, Q3) ^d^			
WBC, per μL	44 (11, 220)	63 (22, 154)	0.34
RBC, per μL	55 (11, 605)	105 (22, 743)	0.29
**Serum biochemical profiles, median** (Q1, Q3) ^d^			
Serum WBC, 10 ^3^ per μL	10.73 (7.55, 15.7)	9.51 (6.51, 13.87)	0.15
Serum ESR, mm/hr	66.5 (38, 92)	55.5 (30, 97.5)	0.63
Troponin I, ng/mL	0.15 (0.04, 0.49)	0.07 (0.04, 0.16)	0.11
Neutrophil, %	78.35 (67.6, 86.1)	78.95 (69.2, 85.05)	0.98
Lymphocyte, %	11.0 (6.05, 17.3)	11.7 (7.00, 21.8)	0.51
NLR	7.00 (3.90, 14.2)	6.91 (3.50, 11.8)	0.61
hs-CRP, mg/dL	6.96 (2.76, 14.2)	6.64 (1.55, 13.5)	0.64
**Mortality**			
In-hospital mortality	73 (24.5)	9 (23.68)	0.91
30-day mortality	53 (17.79)	7 (18.42)	0.92
90-day mortality	79 (26.51)	10 (26.32)	0.98
1-year mortality	113 (37.92)	19 (50)	0.15

CRP, C-reactive protein; ESR, erythrocyte sedimentation rate; IE, infective endocarditis; NLR, neutrophil-lymphocyte ratio; RBC, red blood cell; Q1, 1^st^ quartile; Q3, 3^rd^ quartile; WBC, white blood cell.

a Diagnosis codes that were documented within 1 year of IE diagnosis.

b Valve replacement surgery within 30 days of IE diagnosis.

c Typical pathogens for IE include *Staphylococcus* spp., *S. aureus*, BGS (bovis group streptococci), *S. gallolyticus*, VGS (viridans group streptococci), *Anginosus* group, *S. anginosis*, *S. intermedius*, *Enterococcus* spp., *E. faecium*, *E. faecalis*, *Gemella* spp., *S. morbillorum* (*G. morbillorum*), *Mitis* group, *S. mitis*, *S. oralis, S. sanguinis*, *Mutans* group, *S. mutans*, *Salivarius* group, *S. salivarius*, HACEK group (*H. para-influenzae*, *A. aphrophilus*, *A. ctinomycetemcomitans*, *C. hominis*, *E. corrodens*, *K. denitrificans*, *K. kingae*.

d Serum biochemical profile and urinalyses were performed at the time closest to IE diagnosis.









## Figures and Tables

**Table 1:** Demographic and clinical characteristics of patients screened for infective endocarditis (N = 593).

Variables	IE status according to modified Duke criteria		P value

	Definite or Possible N = 336 (%)	Rejected N = 257 (%)	
**Age** (year, median [Q1, Q3])	60.0 (46.4, 73.2)	70.5 (54.1, 80.5)	<0.0001
18–64 years	200 (59.5)	103 (40.1)	
≥65 years	136 (40.5)	154 (59.9)	
**Male**	203 (60.4)	139 (54.1)	0.122
**Comorbidities** a			
Congestive heart failure	94 (28.0)	91 (35.4)	0.0529
Hypertension	114 (33.9)	112 (43.6)	0.0165
Diabetes mellitus	110 (32.7)	63 (24.5)	0.029
Atrial fibrillation	60 (17.9)	82 (31.9)	<0.0001
Chronic liver disease	40 (11.9)	17 (6.61)	0.0303
Chronic kidney disease	91 (27.1)	47 (18.3)	0.012
Peripheral vascular disease	12 (3.57)	6 (2.33)	0.3843
**Duke criteria**			<0.0001
2 major	173 (51.5)	0 (0)	
1 major and 3–5 minor	51 (15.2)	0 (0)	
0 major and 5 minor	-	-	
1 major and 1–2 minor	96 (28.6)	0 (0)	
0 major and 3–4 minor	16 (4.76)	0 (0)	
0 major and 0–2 minor	0 (0)	257 (100)	
**Valve replacement surgery** b	57 (17.0)	6 (2.33)	<0.0001
**Days from admission to diagnosis**, median (Q1–Q3)	8.00 (1.00, 25.5)	4.00 (1.00, 10.0)	<0.0001
**Blood culture**			
Two positive cultures within 14 days following IE diagnosis	238 (70.8)	37 (14.4)	<0.0001
Two positive cultures with typical pathogens^c^	196 (58.3)	0 (0)	<0.0001
**Sonographic evidence of vegetation**	297 (88.4)	0 (0)	<0.0001
**Fever (≥ 38°C)**	177 (60.8)	65 (29.3)	<0.0001
**Urinalysis**, median (Q1, Q3)^d^			
WBC, per μL	47.0 (14.4, 206)	27.5 (9.00, 160)	0.07
RBC, per μL	63.3 (11.0, 624)	27.5 (5.50, 105)	0.002
**Serum biochemical profiles, median** (Q1, Q3)^d^			
Serum WBC, 10^3^ per μL	10.7 (7.52, 15.2)	7.76 (5.90, 11.0)	<0.0001
Serum ESR, mm/hr	65.5 (36.0, 95.0)	44.0 (20.0, 77.0)	0.006
Troponin I, ng/mL	0.13 (0.04, 0.43)	0.05 (0.02, 0.15)	<0.0001
Neutrophil, %	78.5 (67.9, 86.0)	73.2 (62.6, 82.7)	0.0009
Lymphocyte, %	11.0 (6.30, 17.6)	16.9 (10.6, 23.1)	<0.0001
NLR	7.00 (3.80, 13.6)	4.09 (2.60, 7.35)	<0.0001
hs-CRP, mg/dL	6.96 (2.71, 13.9)	2.81 (0.50, 7.14)	<0.0001
**Mortality**			
In-hospital mortality	82 (24.40)	21 (8.17)	<0.0001
30-day mortality	60 (17.86)	23 (8.95)	0.002
90-day mortality	89 (26.49)	33 (12.84)	<0.0001
1-year mortality	132 (39.29)	55 (21.40)	<0.0001

CRP, C-reactive protein; ESR, erythrocyte sedimentation rate; IE, infective endocarditis; NLR, neutrophil–lymphocyte ratio; RBC, red blood cell; Q1, first quartile; Q3, third quartile; WBC, white blood cell.

a Diagnosis codes that were documented within 1 year prior to IE diagnosis.

b Valve replacement surgery within 30 days of IE diagnosis.

c Typical pathogens for IE include *Staphylococcus* spp., *S. aureus*, BGS (bovis group streptococci), *S. gallolyticus*, VGS (viridans group streptococci), *Anginosus* group, *S. anginosis*, *S. intermedius*, *Enterococcus* spp., *E. faecium*, *E. faecalis*, *Gemella* spp., *S. morbillorum* (*G. morbillorum*), *Mitis* group, *S. mitis*, *S. oralis, S. sanguinis*, *Mutans* group, *S. mutans*, *Salivarius* group, *S. salivarius*, HACEK group (*H. para-influenzae*, *A. aphrophilus*, *A. ctinomycetemcomitans*, *C. hominis*, *E. corrodens*, *K. denitrificans*, *K. kingae*.

d Serum biochemical profile and urinalysis were performed at the time closest to IE diagnosis.

**Table 2:** Comparison of positive predictive value and age-adjusted in-hospital mortality according to different case identification strategies.

Case identification strategies	Sample size	PPV	Crude mortality (%)	Age-adjusted in-hospital mortality^a^
ICD	593	0.57 (0.53–0.61)	17.4	15.9
ICD and (Fever or PBC or Vegetation)	373	0.78 (0.73–0.82)	20.9	19.4
ICD and (Fever or PBC)	368	0.76 (0.71–0.80)	21.7	19.8
ICD and (PBC or Vegetation)	363	0.90 (0.86–0.93)	24.5	21.8
ICD and (Fever or Vegetation)	347	0.81 (0.77–0.85)	21.6	19.9
**ICD and Duke-confirmed by chart review (Reference standard)** b	336	-	24.4	21.0
ICD and Vegetation	297	1.00 (0.99–1.00)	24.9	21.5
ICD and PBC	275	0.87 (0.82–0.90)	25.8	22.9
ICD and Fever	242	0.73 (0.67–0.79)	21.1	19.8
ICD and (PBC and Vegetation)	209	1.00 (0.98–1.00)	26.8	22.7
ICD and (Fever and PBC)	149	0.92 (0.86–0.96)	28.2	25.7
ICD and (Fever and Vegetation)	149	1.00 (0.98–1.00)	25.5	23.0
ICD and (Fever and PBC and Vegetation)	118	1.00 (0.97–1.00)	27.1	24.4

ICD, International Classification of Diseases; PBC, positive blood culture; PPV, positive predictive value.

a Mortality was adjusted by age using logistic regression.

b Chart review was performed using the Duke criteria and definite or possible cases were considered.

**Table 3:** Comparison of positive predictive value and age-adjusted in-hospital mortality according to more sensitive case identification strategies by excluding ICD-9 424.9 or ICD-10 I38.

Case identification strategies	Sample size	PPV	Crude mortality (%)	Age-adjusted in-hospital mortality^a^
ICD	358	0.83 (0.79–0.87)	22.9	19.9
ICD and (PBC or Vegetation)	312	0.94 (0.91–0.97)	25.0	21.8
**ICD and Duke-confirmed by chart review (Reference standard)** b	298	-	24.5	21.1
ICD and (Fever or PBC or Vegetation)	283	0.90 (0.86–0.93)	23.0	20.6
ICD and (Fever or PBC)	278	0.88 (0.84–0.92)	24.5	21.3
ICD and Vegetation	273	1.00 (0.99–1.00)	24.5	21.0
ICD and (Fever or Vegetation)	267	0.93 (0.89–0.95)	24.0	21.4
ICD and PBC	234	0.92 (0.88–0.95)	26.9	23.2
ICD and (PBC and Vegetation)	195	1.00 (0.98–1.00)	26.7	22.3
ICD and Fever	171	0.88 (0.83–0.93)	25.2	23.0
ICD and (Fever and Vegetation)	136	1.00 (0.97–1.00)	25.0	22.5
ICD and (Fever and PBC)	127	0.95 (0.90–0.98)	29.9	27.4
ICD and (Fever and PBC and Vegetation)	109	1.00 (0.97–1.00)	27.5	24.6

ICD, International Classification of Diseases; PBC, positive blood culture; PPV, positive predictive value.

a Mortality was adjusted by age using logistic regression.

b Chart review was performed using the Duke criteria and definite or possible cases were considered.
