# Supplementary material for: Electronic medical record-based deep data cleaning and phenotyping improve the diagnostic validity and mortality assessment of infective endocarditis: medical big data initiative of CMUH
Source: Biomedicine (Taipei). 2021 Sep 1;11(3):59–67. doi: 10.37796/2211-8039.1267 (PMC8823496; doi:10.37796/2211-8039.1267)
Supplement: Supplementary file 1 [file bmed-11-03-059-s001.docx]

**Electronic Medical Record-Based Deep Data Cleaning and**

**Phenotyping Improve the Diagnostic Validity and Mortality**

**Assessment of Infective Endocarditis: Medical Big Data Initiative**

**of CMUH**

Hsiu-Yin Chiang, MS, PhD^1^; Li-Ying Liang, MD^2^; Che-Chen Lin, MS^1^; Yi-Jin Chen, MS^3^; Min-Yen Wu, MS^1^; Sheng-Hsuan Chen, MS^1^; Pin-Hua Wu, MS^4^; Chin-Chi Kuo, MD, PhD^1,3,5,6^; Chih-Yu Chi, MD, PhD^2,6,*^

^1^Big Data Center, China Medical University Hospital, Taichung, Taiwan; ^2^Division of Infectious Diseases, Department of Internal Medicine, China Medical University Hospital, Taichung, Taiwan; ^3^Department of Medical Research, China Medical University Hospital, Taichung, Taiwan; ^4^Department of Computer Science, National Tsing-Hua University; Hsinchu, Taiwan; ^5^Kidney Institute and Division of Nephrology, Department of Internal Medicine, China Medical University Hospital, Taichung, Taiwan; ^6^College of Medicine, China Medical University, Taichung Taiwan.

Running title: Using EMR to identify infective endocarditis.

Word counts: Abstract 253 words; Text 2365 words.

Conflict of interest statement: All authors declare no conflict of interest.

Corresponding author:

*Chih-Yu Chi, MD, PhD. Division of Infectious Diseases, Department of Internal Medicine, China Medical University Hospital.

Add: No. 2 Yu-Der Rd, North Dist., Taichung City 404, Taiwan

Tel: +886-4-822052121 ext. 1870

Fax: +886-4-2207-5869

E-mail: [cychyi@gmail.com](mailto:cychyi@gmail.com)

**ABSTRACT**

**Background**

International Classification of Diseases (ICD) code–based claims databases are often used to study infective endocarditis (IE). However, the quality of ICD coding can influence the reliability of IE research. The impact of complementing the ICD-only approach with data extracted from electronic medical records (EMRs) has yet to be explored.

**Methods**

We selected the information of adult patients with discharge ICD codes for IE (ICD-9: 421, 112.81, 036.42, 098.84, 115.04, 115.14, 115.94, 424.9; ICD-10: I33, I38, I39) during 2005–2016 in China Medical University Hospital. Data extraction was conducted on the basis of the modified Duke criteria to establish a reference group comprising patients with definite or possible IE. Clinical characteristics and in-hospital mortality were compared between ICD-identified and Duke-confirmed cases. The positive predictive value (PPV) was used to quantify the IE identification performance of various phenotyping algorithms.

**Results**

A total of 593 patients with discharge ICD codes for IE were identified, only 56.7% met the modified Duke criteria. The crude in-hospital mortality for Duke-confirmed and Duke-rejected IE were 24.4% and 8.2%, respectively. The adjusted in-hospital mortality for ICD-identified IE was lower than that for Duke-confirmed IE by a difference of 5.1%. The best PPV was achieved (0.90, 95% CI 0.86–0.93) when major components of the Duke criteria (positive blood culture and vegetation) were integrated with ICD codes.

**Conclusion**

Integrating EMR data can considerably improve the accuracy of ICD-only approaches in phenotyping IE, which can improve the validity of EMR-based studies and their applications, including real-time surveillance and clinical decision support.

**Keywords:** Disease phenotyping; electronic medical record; infective endocarditis; International Classification of Diseases; positive predictive value.
