# Supplementary material for: Electronic medical record-based deep data cleaning and phenotyping improve the diagnostic validity and mortality assessment of infective endocarditis: medical big data initiative of CMUH
Source: Biomedicine (Taipei). 2021 Sep 1;11(3):59–67. doi: 10.37796/2211-8039.1267 (PMC8823496; doi:10.37796/2211-8039.1267)
Supplement: Supplementary file 2 [file bmed-11-03-059-s002.docx]

**Authorship statement**

HYC, CCK, and CYC designed the study. CCL, LYL, and YJC performed data quality management and statistical analysis. MYW, SHC, and PHW conducted natural language processing of microbiology text reports. HYC and CYC drafted the manuscript. HYC, CCL, CCK, and CYC critically edited the manuscript. All authors read and approved the final manuscript.

**Funding statement**

This study was partially funded by the Ministry of Science and Technology (MOST), Taiwan and China Medical University Hospital (CMUH), Taichung, Taiwan (MOST grant: 108-2314-B-039-038-MY3 & 109-2321-B-468-001; CMUH grant: DMR-110- 001, DMR-HHC-110-1, DMR-HHC-110-2).

**Acknowledgments**

We appreciate the data exploration, statistical analysis, manuscript preparation, and the support of the iHi Clinical Research Platform from the Big Data Center of CMUH. We would like to thank the Health and Welfare Data Science Center (HWDC), Ministry of Health Welfare, and Health Data Science Center, China Medical University Hospital for providing administrative, technical, and funding support.
