# Supplementary material for: Electronic medical record-based deep data cleaning and phenotyping improve the diagnostic validity and mortality assessment of infective endocarditis: medical big data initiative of CMUH
Source: Biomedicine (Taipei). 2021 Sep 1;11(3):59–67. doi: 10.37796/2211-8039.1267 (PMC8823496; doi:10.37796/2211-8039.1267)
Supplement: Supplementary file 3 [file bmed-11-03-059-s003.docx]

**Table 1.** Demographic and clinical characteristics of patients screened for infective endocarditis (*N* = 593).

|  | **IE status according to modified Duke criteria** | |  |
| --- | --- | --- | --- |
| **Variables** | Definite or Possible  N = 336 (%) | Rejected  N = 257 (%) | P value |
| **Age** (year, median [Q1, Q3]) | 60.0 (46.4, 73.2) | 70.5 (54.1, 80.5) | <0.0001 |
| 18-64 years | 200 (59.5) | 103 (40.1) |  |
| ≥65 years | 136 (40.5) | 154 (59.9) |  |
| **Male** | 203 (60.4) | 139 (54.1) | 0.122 |
| **Comorbidities** ^a^ |  |  |  |
| Congestive heart failure | 94 (28.0) | 91 (35.4) | 0.0529 |
| Hypertension | 114 (33.9) | 112 (43.6) | 0.0165 |
| Diabetes mellitus | 110 (32.7) | 63 (24.5) | 0.029 |
| Atrial fibrillation | 60 (17.9) | 82 (31.9) | <0.0001 |
| Chronic liver disease | 40 (11.9) | 17 (6.61) | 0.0303 |
| Chronic kidney disease | 91 (27.1) | 47 (18.3) | 0.012 |
| Peripheral vascular disease | 12 (3.57) | 6 (2.33) | 0.3843 |
| **Duke criteria** |  |  | <0.0001 |
| 2 major | 173 (51.5) | 0 (0) |  |
| 1 major and 3-5 minor | 51 (15.2) | 0 (0) |  |
| 0 major and 5 minor | - | - |  |
| 1 major and 1-2 minor | 96 (28.6) | 0 (0) |  |
| 0 major and 3-4 minor | 16 (4.76) | 0 (0) |  |
| 0 major and 0-2 minor | 0 (0) | 257 (100) |  |
| **Valve replacement surgery** ^b^ | 57 (17.0) | 6 (2.33) | <0.0001 |
| **Days from admission to diagnosis**, median (Q1-Q3) | 8.00 (1.00, 25.5) | 4.00 (1.00, 10.0) | <0.0001 |
| **Blood culture** |  |  |  |
| Two positive cultures within 14 days following IE diagnosis | 238 (70.8) | 37 (14.4) | <0.0001 |
| Two positive cultures with typical pathogens ^c^ | 196 (58.3) | 0 (0) | <0.0001 |
| **Sonographic evidence of vegetation** | 297 (88.4) | 0 (0) | <0.0001 |
| **Fever (≥ 38^o^C)** | 177 (60.8) | 65 (29.3) | <0.0001 |
| **Urinalysis**, median (Q1, Q3) ^d^ |  |  |  |
| WBC, per μL | 47.0 (14.4, 206) | 27.5 (9.00, 160) | 0.07 |
| RBC, per μL | 63.3 (11.0, 624) | 27.5 (5.50, 105) | 0.002 |
| **Serum biochemical profiles, median** (Q1, Q3) ^d^ |  |  |  |
| Serum WBC, 10^3^ per μL | 10.7 (7.52, 15.2) | 7.76 (5.90, 11.0) | <0.0001 |
| Serum ESR, mm/hr | 65.5 (36.0, 95.0) | 44.0 (20.0, 77.0) | 0.006 |
| Troponin I, ng/mL | 0.13 (0.04, 0.43) | 0.05 (0.02, 0.15) | <0.0001 |
| Neutrophil, % | 78.5 (67.9, 86.0) | 73.2 (62.6, 82.7) | 0.0009 |
| Lymphocyte, % | 11.0 (6.30, 17.6) | 16.9 (10.6, 23.1) | <0.0001 |
| NLR | 7.00 (3.80, 13.6) | 4.09 (2.60, 7.35) | <0.0001 |
| hs-CRP, mg/dL | 6.96 (2.71, 13.9) | 2.81 (0.50, 7.14) | <0.0001 |
| **Mortality** |  |  |  |
| In-hospital mortality | 82 (24.40) | 21 (8.17) | <0.0001 |
| 30-day mortality | 60 (17.86) | 23 (8.95) | 0.002 |
| 90-day mortality | 89 (26.49) | 33 (12.84) | <0.0001 |
| 1-year mortality | 132 (39.29) | 55 (21.40) | <0.0001 |

CRP, C-reactive protein; ESR, erythrocyte sedimentation rate; IE, infective endocarditis; NLR, neutrophil–lymphocyte ratio; RBC, red blood cell; Q1, first quartile; Q3, third quartile; WBC, white blood cell.

^a^Diagnosis codes that were documented within 1 year prior to IE diagnosis.

^b^Valve replacement surgery within 30 days of IE diagnosis.

^c^Typical pathogens for IE include *Staphylococcus* spp., *S. aureus*, BGS (bovis group streptococci), *S. gallolyticus*, VGS (viridans group streptococci), *Anginosus* group, *S. anginosis*, *S. intermedius*, *Enterococcus* spp., *E. faecium*, *E. faecalis*, *Gemella* spp., *S. morbillorum* (*G. morbillorum*), *Mitis* group, *S. mitis*, *S. oralis, S. sanguinis*, *Mutans* group, *S. mutans*, *Salivarius* group, *S. salivarius*, HACEK group (*H. parainfluenzae*, *A. aphrophilus*, *A. ctinomycetemcomitans*, *C. hominis*, *E. corrodens*, *K. denitrificans*, *K. kingae*.

^d^Serum biochemical profile and urinalysis were performed at the time closest to IE diagnosis.

**Table 2.** Comparison of positive predictive value and age-adjusted in-hospital mortality according to different case identification strategies.

| **Case identification strategies** | **Sample size** | **PPV** | **Crude mortality (%)** | **Age-adjusted**  **in-hospital mortality ^a^** |
| --- | --- | --- | --- | --- |
| ICD | 593 | 0.57 (0.53-0.61) | 17.4 | 15.9 |
| ICD and (Fever or PBC or Vegetation) | 373 | 0.78 (0.73-0.82) | 20.9 | 19.4 |
| ICD and (Fever or PBC) | 368 | 0.76 (0.71-0.80) | 21.7 | 19.8 |
| ICD and (PBC or Vegetation) | 363 | 0.90 (0.86-0.93) | 24.5 | 21.8 |
| ICD and (Fever or Vegetation) | 347 | 0.81 (0.77-0.85) | 21.6 | 19.9 |
| **ICD and Duke-confirmed by chart review (Reference standard)^b^** | 336 | - | 24.4 | 21.0 |
| ICD and Vegetation | 297 | 1.00 (0.99-1.00) | 24.9 | 21.5 |
| ICD and PBC | 275 | 0.87 (0.82-0.90) | 25.8 | 22.9 |
| ICD and Fever | 242 | 0.73 (0.67-0.79) | 21.1 | 19.8 |
| ICD and (PBC and Vegetation) | 209 | 1.00 (0.98-1.00) | 26.8 | 22.7 |
| ICD and (Fever and PBC) | 149 | 0.92 (0.86-0.96) | 28.2 | 25.7 |
| ICD and (Fever and Vegetation) | 149 | 1.00 (0.98-1.00) | 25.5 | 23.0 |
| ICD and (Fever and PBC and Vegetation) | 118 | 1.00 (0.97-1.00) | 27.1 | 24.4 |

ICD, International Classification of Diseases; PBC, positive blood culture; PPV, positive predictive value.

^a^Mortality was adjusted by age using logistic regression.

^b^Chart review was performed using the Duke criteria and definite or possible cases were considered.

**Table 3.** Comparison of positive predictive value and age-adjusted in-hospital mortality according to more sensitive case identification strategies by excluding ICD-9 424.9 or ICD-10 I38.

| **Case identification strategies** | **Sample size** | **PPV** | **Crude mortality (%)** | **Age-adjusted**  **in-hospital mortality ^a^** |
| --- | --- | --- | --- | --- |
| ICD | 358 | 0.83 (0.79-0.87) | 22.9 | 19.9 |
| ICD and (PBC or Vegetation) | 312 | 0.94 (0.91-0.97) | 25.0 | 21.8 |
| **ICD and Duke-confirmed by chart review (Reference standard)^b^** | 298 | - | 24.5 | 21.1 |
| ICD and (Fever or PBC or Vegetation) | 283 | 0.90 (0.86-0.93) | 23.0 | 20.6 |
| ICD and (Fever or PBC) | 278 | 0.88 (0.84-0.92) | 24.5 | 21.3 |
| ICD and Vegetation | 273 | 1.00 (0.99-1.00) | 24.5 | 21.0 |
| ICD and (Fever or Vegetation) | 267 | 0.93 (0.89-0.95) | 24.0 | 21.4 |
| ICD and PBC | 234 | 0.92 (0.88-0.95) | 26.9 | 23.2 |
| ICD and (PBC and Vegetation) | 195 | 1.00 (0.98-1.00) | 26.7 | 22.3 |
| ICD and Fever | 171 | 0.88 (0.83-0.93) | 25.2 | 23.0 |
| ICD and (Fever and Vegetation) | 136 | 1.00 (0.97-1.00) | 25.0 | 22.5 |
| ICD and (Fever and PBC) | 127 | 0.95 (0.90-0.98) | 29.9 | 27.4 |
| ICD and (Fever and PBC and Vegetation) | 109 | 1.00 (0.97-1.00) | 27.5 | 24.6 |

ICD*,* International Classification of Diseases; PBC, positive blood culture; PPV, positive predictive value.

^a^Mortality was adjusted by age using logistic regression.

^b^Chart review was performed using the Duke criteria and definite or possible cases were considered.
