# Supplementary material for: Electronic medical record-based deep data cleaning and phenotyping improve the diagnostic validity and mortality assessment of infective endocarditis: medical big data initiative of CMUH
Source: Biomedicine (Taipei). 2021 Sep 1;11(3):59–67. doi: 10.37796/2211-8039.1267 (PMC8823496; doi:10.37796/2211-8039.1267)
Supplement: Supplementary file 4 [file bmed-11-03-059-s004.docx]

**Supplemental Table 1**. International Classification of Diseases, 9^th^ Revision, Clinical Modification (ICD-9-CM) diagnosis codes and ICD-10-CM diagnosis codes for defining comorbidities within 1 year of infective endocarditis diagnosis.

| **Comorbidities** | **ICD-9-CM** | **ICD-10-CM** |
| --- | --- | --- |
| Congestive heart failure | 398.91, 402.01, 402.11, 402.91, 404.01, 404.03, 404.11, 404.13, 404.91, 404.93, 425.4-425.9, 428.x | I09.9, I11.0, I13.0, I13.2, I25.5, I42.0,  I42.5-I42.9, I43.x, I50.x, P29.0 |
| Diabetes mellitus | 250.0-250.3, 250.8, 250.9, 250.4-250.7 | E10.0, E10.l, E10.6, E10.8, E10.9, E11.0, E11.1, E11.6, E11.8, E11.9, E12.0, E12.1, E12.6, E12.8, E12.9, E13.0, E13.1, E13.6, E13.8, E13.9, E14.0, E14.1, E14.6, E14.8, E14.9, E10.2-E10.5, E10.7, E11.2-E11.5, E11.7, E12.2-E12.5, E12.7, E13.2-E13.5, E13.7, E14.2-E14.5, E14.7 |
| Chronic liver disease | 070.22, 070.23, 070.32, 070.33, 070.44, 070.54, 070.6, 070.9, 570.x, 571.x, 573.3, 573.4, 573.8, 573.9, V42.7, 456.0-456.2, 572.2-572.8 | B18.x, K70.0-K70.3, K70.9, K71.3-K71.5, K71.7, K73.x, K74.x,  K76.0, K76.2-K76.4, K76.8, K76.9, Z94.4, I85.0, I85.9, I86.4, I98.2, K70.4, K71.1, K72.1, K72.9, K76.5, K76.6, K76.7 |
| Hypertension | 401-405 | I10-I15 |
| Peripheral vascular disease | 093.0, 437.3, 440.x, 441.x, 443.1-443.9, 447.1, 557.1, 557.9, V43.4 | I70.x, I71.x, I73.1, I73.8, I73.9, I77.1, I79.0, I79.2, K55.1, K55.8, K55.9, Z95.8, Z95.9 |
| Chronic kidney disease | 582, 585, 586, 588, 583.0-583.7  ESRD 585 (Catastrophic illness) | ESRD: N18.5, N18.6, I12.0, I13.2, I13.11 |

**Supplementary Table 2.** Demographic and clinical characteristics of patients with infective endocarditis confirmed on the basis of Duke criteria (definite or possible).

|  | **Patients with Duke-confirmed IE (N = 336)** | |  |
| --- | --- | --- | --- |
| **Variables** | With ICD of  424.9 or I38  N = 298 (88.7%) | Without ICD of  424.9 or I38  N = 38 (11.3%) | P value |
| **Age** (year, median [Q1, Q3]) | 59.1 (46.22, 72.16) | 71.79 (52.46, 79.13) |  |
| 18-64 years | 186 (62.42) | 14 (36.84) | 0.003 |
| ≥65 years | 112 (37.58) | 24 (63.16) |  |
| **Male** | 183 (61.41) | 20 (52.63) | 0.30 |
| **Comorbidities** ^a^ |  |  |  |
| Congestive heart failure | 79 (26.51) | 15 (39.47) | 0.09 |
| Hypertension | 100 (33.56) | 14 (36.84) | 0.69 |
| Diabetes mellitus | 102 (34.23) | 8 (21.05) | 0.10 |
| Atrial fibrillation | 54 (18.12) | 6 (15.79) | 0.72 |
| Chronic liver disease | 37 (12.42) | 3 (7.89) | 0.42 |
| Chronic kidney disease | 82 (27.52) | 9 (23.68) | 0.62 |
| Peripheral vascular disease | 10 (3.36) | 2 (5.26) | 0.55 |
| **Duke criteria** |  |  | <0.0001 |
| 2 major | 159 (53.36) | 14 (36.84) |  |
| 1 major and 3-5 minor | 46 (15.44) | 5 (13.16) |  |
| 0 major and 5 minor | - | - |  |
| 1 major and 1-2 minor | 85 (28.52) | 11 (28.95) |  |
| 0 major and 3-4 minor | 8 (2.68) | 8 (21.05) |  |
| 0 major and 0-2 minor | - | - |  |
| **Valve replacement surgery** ^b^ | 53 (17.79) | 4 (10.53) | 0.26 |
| **Days from admission to diagnosis**, median (Q1-Q3) | 8 (1, 27) | 9 (1, 23) |  |
| **Blood culture** |  |  |  |
| Two positive cultures within 14 days following IE diagnosis | 216 (72.48) | 22 (57.89) | 0.06 |
| Two positive cultures with typical pathogens ^c^ | 176 (59.06) | 20 (52.63) | 0.45 |
| **Sonographic evidence of vegetation** | 273 (91.61) | 24 (63.16) | <0.0001 |
| **Fever (≥ 38^o^C)** | 151 (50.67) | 26 (68.42) | 0.04 |
| **Urinalysis**, median (Q1, Q3) ^d^ |  |  |  |
| WBC, per μL | 44 (11, 220) | 63 (22, 154) | 0.34 |
| RBC, per μL | 55 (11, 605) | 105 (22, 743) | 0.29 |
| **Serum biochemical profiles, median** (Q1, Q3) ^d^ |  |  |  |
| Serum WBC, 10^3^ per μL | 10.73 (7.55, 15.7) | 9.51 (6.51, 13.87) | 0.15 |
| Serum ESR, mm/hr | 66.5 (38, 92) | 55.5 (30, 97.5) | 0.63 |
| Troponin I, ng/mL | 0.15 (0.04, 0.49) | 0.07 (0.04, 0.16) | 0.11 |
| Neutrophil, % | 78.35 (67.6, 86.1) | 78.95 (69.2, 85.05) | 0.98 |
| Lymphocyte, % | 11.0 (6.05, 17.3) | 11.7 (7.00, 21.8) | 0.51 |
| NLR | 7.00 (3.90, 14.2) | 6.91 (3.50, 11.8) | 0.61 |
| hs-CRP, mg/dL | 6.96 (2.76, 14.2) | 6.64 (1.55, 13.5) | 0.64 |
| **Mortality** |  |  |  |
| In-hospital mortality | 73 (24.5) | 9 (23.68) | 0.91 |
| 30-day mortality | 53 (17.79) | 7 (18.42) | 0.92 |
| 90-day mortality | 79 (26.51) | 10 (26.32) | 0.98 |
| 1-year mortality | 113 (37.92) | 19 (50) | 0.15 |

CRP, C-reactive protein; ESR, erythrocyte sedimentation rate; IE, infective endocarditis; NLR, neutrophil-lymphocyte ratio; RBC, red blood cell; Q1, 1^st^ quartile; Q3, 3^rd^ quartile; WBC, white blood cell.

^a^Diagnosis codes that were documented within 1 year of IE diagnosis.

^b^Valve replacement surgery within 30 days of IE diagnosis.

^c^Typical pathogens for IE include *Staphylococcus* spp., *S. aureus*, BGS (bovis group streptococci), *S. gallolyticus*, VGS (viridans group streptococci), *Anginosus* group, *S. anginosis*, *S. intermedius*, *Enterococcus* spp., *E. faecium*, *E. faecalis*, *Gemella* spp., *S. morbillorum* (*G. morbillorum*), *Mitis* group, *S. mitis*, *S. oralis, S. sanguinis*, *Mutans* group, *S. mutans*, *Salivarius* group, *S. salivarius*, HACEK group (*H. parainfluenzae*, *A. aphrophilus*, *A. ctinomycetemcomitans*, *C. hominis*, *E. corrodens*, *K. denitrificans*, *K. kingae*.

^d^Serum biochemical profile and urinalyses were performed at the time closest to IE diagnosis.
